# The Mediating Role of Teacher Efficacy Between Academic Self-Concept and Teacher Identity Among Pre-Service Physical Education Teachers: Is There a Gender Difference?

**DOI:** 10.3390/bs14111053

**Published:** 2024-11-06

**Authors:** José María Rubio-Valdivia, Antonio Granero-Gallegos, María Carrasco-Poyatos, Ginés David López-García

**Affiliations:** 1 Department of Education, University of Almeria, 04120 Almeria, Spain; josemariarubio98@gmail.com (J.M.R.-V.); carrasco@ual.es (M.C.-P.); 2 Health Research Centre, University of Almeria, 04120 Almeria, Spain; 3 Department of Plastic, Musical and Dynamic Expression, University of Murcia, 30100 Murcia, Spain; glopez@um.es

**Keywords:** academic confidence, professional identity, teaching identity, teacher education

## Abstract

The aim of this study was to analyse the differentiated role played by academic self-concept on teacher identity, taking into account the mediating role of teacher efficacy and the gender of the pre-service physical-education teachers. In this cross-sectional study, 478 master’s degree students in Secondary Education Teacher Training participated (M_ean_ = 27.09; SD = 6.32; 54.8% male, 44.8% female, 0.4% other). The following scales were used: academic self-concept, teaching identity, and teacher self-efficacy. After finding significant differences in academic effort, a structural equation analysis (SEM) with the multigroup option was conducted to answer the research objective. The results revealed that the women had significantly higher mean academic effort values than the men. In addition, the SEM, which showed excellent fit indices, revealed that, for the men, teacher efficacy acted as a mediating variable between academic confidence and teacher identity. In contrast, for the women, teacher efficacy acted as a mediator between academic effort and teacher identity. However, academic effort also acted as a direct predictor of teacher identity in male pre-service teachers. As a main conclusion, it can be said that the findings highlight the importance of academic self-concept in undertaking the master’s degree by increasing teacher identity through the teaching efficacy of pre-service teacher educators.

## 1. Introduction

For more than two decades, studies on teacher education programmes have been carried out to explore the process of forming professional identity in students [1,2,3]. These teacher training programmes are considered the first stage, and one of the most important, in developing teaching identity in pre-service teachers [2]. However, despite the scientific evidence in this area [1,2,3], few studies have rigorously contributed to understanding how the self-regulating adjustments of pre-service teachers influence teacher identity during the teacher training programme. Therefore, a better conceptual understanding of the pre-service teachers’ teaching identity could contribute significantly to the efficient design of this initial training [4]. The scientific literature covering the education field has examined the influence of variables such as previous experiences [5] and context [6] on the process of forming the students’ professional identity as well as intrinsic resources such as academic self-concept [7]. Each of these variables seems to have (un)favourable repercussions on the pre-service teachers’ identity during training; for this reason, it is important to study their influence.

### 1.1. Teaching Identity

Teaching identity has been conceptualized as a theoretical construct that encompasses activities, identification, and categorization processes, through which we situate and position ourselves as teachers in front of other individuals [8]. In this regard, a strong professional identity is linked to classroom teaching quality [9], commitment to the teaching profession, and improved self-confidence as teachers [10]. Moreover, the process of constructing teaching identity has traditionally been studied by identifying how it forms gradually throughout the subject’s schooling up to their own professional development [11]. However, authors such as Sánchez and González de Álvarez [12] have shown that teaching identity begins to consolidate during the student’s pedagogical training. In this way, there are several necessary scenarios that create teaching identity in pedagogical training programmes (e.g., socially localized influences, personal influences, etc.) [13]. Among these, personal influences are developed by self-perceptions and intrinsic resources innate to the person, such as self-concept or teaching effectiveness, which form teaching identity [14,15].

### 1.2. Teaching Sense of Efficacy

One of the most widely used theories that supports effectiveness in the teaching field is the Social Cognitive Theory (SCT; Bandura) [16,17]. According to the postulates of this theory, individuals are capable of acting as causal agents in the actions that determine their future existence. Likewise, Bandura [17] postulates that those individuals who perceive themselves as efficient will set themselves higher personal challenges and have higher levels of personal commitment towards these objectives. In the context of teacher education, teaching effectiveness refers to a pre-service teacher’s beliefs regarding their own ability to successfully perform in the teaching profession [16]. In this way, pre-service teachers who perceive themselves as efficient will tackle challenging activities and show greater commitment during training activities, thus increasing their well-being and positive development [18]. Confirming the theoretical approaches of the SCT, some research has shown that the teacher’s sense of efficacy has implications for cognitive and motivational processes during the teacher training process [18,19,20]. Indeed, recent research has evidenced the relationship between teacher effectiveness and the teaching identity of pre-service teachers [21]; that is to say, perceived self-efficacy focuses on the possibility of achieving future undertakings and the need for competence focuses on achieving a future professional identity, derived from the experience of present competence.

### 1.3. Academic Self-Concept

Academic self-concept has been conceptualized as the set of traits and qualities that a person attributes to themselves in the academic field, constituting their own cognitive representation [22]. At the theoretical level, self-concept has been supported by several theories that have been compared in the educational field [23]. Accordingly, in the university context and in the field of teacher training, self-concept has been conceptualized as a two-dimensional construct categorized into academic confidence and academic effort [24,25]. Academic confidence is understood as the assessment of feelings and perceptions regarding one’s own academic competence. The development of academic confidence leads individuals to undertake learning activities and pursue their goals [26], while low confidence levels can limit academic progress, lower academic performance, or even lead to dropout [27]. On the other hand, academic effort is understood as the individual’s intrinsic willingness to participate in academic work. Academic effort is considered to be an important factor in the processes of adapting to university studies and a promoter of teaching effectiveness [28], while its underdevelopment decreases students’ academic expectations [29]. Despite the small amount of teacher training research having looked at academic effort and academic confidence in Physical Education (PE) trainee teachers, to the best of our knowledge, no study has been carried out that considers the students’ gender.

### 1.4. Gender Differences

The scant previous work that has been carried out in the PE context [30,31] and in PE teacher training [32,33] has led to contradictory findings regarding gender differences in the students’ intrinsic resources (e.g., professional identity, self-concept, and teaching efficacy). For example, some of the previous teacher training studies did not differentiate between men and women in terms of academic self-concept [34,35], teaching self-efficacy [19,20] or teaching identity [36]. However, other studies, such as that by Klassen and Chiu [37] have shown higher levels of teaching self-efficacy in male teachers than in female teachers. On the other hand, with respect to teaching identity, the scientific evidence from the teacher training field has shown that teaching behaviours such as professional identity can vary according to the gender of the individual [36]. Finally, studies such as that by Liu et al. [38] or Matovu [39] have shown that academic self-concept is influenced by variables such as the students’ gender. In this regard, males could present different academic self-concept values to females. This previous work therefore suggests that intrinsic behaviours, such as self-concept, might influence the professional identification process in different ways. However, there are no studies that have examined the extent to which academic confidence or academic effort can trigger different ways of constructing professional identity among students in initial teacher training. From a theoretical perspective, examining gender differences in intrinsic behaviours by considering the SCT postulates can help researchers to better understand these differences between men and women in relation to PE teacher training.

### 1.5. The Present Study

**Figure 1:**
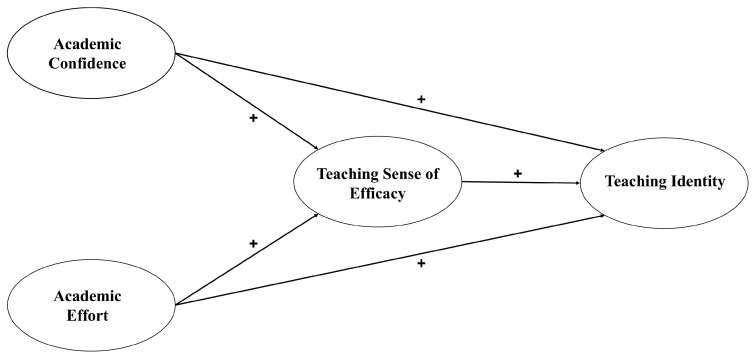
Hypothesized model.

As far as we are aware, this is the first study that analyses the possible influence of academic self-concept on the professional identity of future teachers, taking into account the students’ gender. Given the importance of the above-mentioned gaps in the literature, our research has two main objectives. The first objective seeks to identify possible gender differences in academic self-concept, teacher efficacy, and teaching identity. Secondly, we performed a multi-group path analysis to examine the possible differences in the predictive relationships between the students’ self-concept and teaching identity, taking into account teaching efficacy in initial teacher training amongst girls and boys (Figure 1). Given the absence of a consistent body of prior research, we did not venture to formulate any hypotheses. The ‘Strengthening the Reporting of Observational Studies in Epidemiology’ (STROBE) initiative was used for the study description [40].

## 2. Materials and Methods

### 2.1. Design and Participants

The design of this research is observational, cross-sectional, and descriptive. Students enrolled in the Master’s Degree in Teaching (MAES) from different Andalusian public universities participated in the study. The inclusion criteria to participate in the research were: (i) to have been studying MAES during the 21/22 academic year; (ii) to have regularly attended face-to-face teaching; (iii) to have provided one’s informed consent to participate in the research; and (iv) to have fully completed the questionnaire. In addition, an a priori analysis of the sample size was carried out to meet the study objective. This was conducted using Free Statistics Calculator v.4.0 software [41]. It was calculated that a minimum of 468 teachers in initial training were required to detect effect sizes of f^2^ = 0.19, with a statistical power of 90% and a significance level of α = 0.05 in a structural equation model (SEM) with four latent variables and 18 observable variables. In the end, 478 future teachers in initial teacher training participated in the study.

### 2.2. Measurements

Teachers Professional Identity (IPD). The Spanish version [36] of the Student Teachers Professional Identity Scale by Fisherman and Weiss [42] was used. This one-dimensional scale consists of nine items (e.g., ‘I feel comfortable saying that I will be a teacher’) and assesses the extent to which teachers view their profession as a mission, their confidence in the career choice they have made, and their sense of self-realisation. The responses were collected using a Likert-type scale that ranged from 1 (Strongly disagree) to 5 (Strongly agree). The scale was used as a higher-order model (one-dimensional). The reliability obtained was: ω = 0.92. A Confirmatory Factor Analysis (CFA) of the factor structure was carried out, showing adequate goodness-of-fit indices: χ^2^/df (chi squared/degrees of freedom) = 4.66, *p* < 0.001; Comparative Fit Index (CFI) = 0.97; Tucker–Lewis Index (TLI) = 0.95; Root Mean Square Error of Approximation (RMSEA) = 0.078 [90% Confidence Interval (CI) = 0.066; 0.094]; and Standardized Root Mean Squared Residual (SRMR) = 0.033. The reliability values were acceptable: teacher identity, McDonald’s Omega (ω) = 0.91.

Academic Self-Concept (ASC). The Spanish version [24] of the Academic Self-Concept Scale by Matovu [39] was used. This instrument is composed of three items that measure academic effort (e.g., ‘I pay attention to the professor during classes’) and three items that measure academic confidence (e.g., ‘I can easily follow the flow of the classes’). The responses were collected using a Likert-type scale that ranged from 1 (Completely disagree) to 7 (Completely agree). The reliability obtained was: (i) effort ω = 0.78; and (ii) confidence ω = 0.72. The goodness-of-fit indices for the CFA of this scale were acceptable: χ^2^/df = 4.64, *p* < 0.001; CFI = 0.96; TLI = 0.93; RMSEA = 0.078 (90%CI = 0.057, 0.103); and SRMR = 0.047. The reliability obtained was acceptable: Academic effort, ω = 0.77; Academic confidence, ω = 0.71.

Teaching Sense of Efficacy (SEE). The Spanish version [19] of the Teacher’s Sense of Efficacy by Tschannen-Moran and Hoy [43] was used. This instrument comprises four items that measure teaching strategies (e.g., ‘To what extent could you ask students good questions?’), four items measuring effective student engagement (e.g., ‘How much could you do to help students value their learning?’) and three items measuring classroom management (e.g., ‘How much could you do to control misbehaviour in class?’). The responses were collected in a Likert-type scale which ranged from 1 (not at all) to 9 (a lot). The CFA goodness-of-fit indices of this scale were acceptable: χ^2^/df = 3.87; *p* < 0.001; CFI = 0.97; TLI = 0.96; RMSEA = 0.078 (90%CI = 0.065, 0.90); and SRMR = 0.047. The scale was used as a higher-order (one-dimensional) model. The reliability obtained was: ω = 0.96.

### 2.3. Procedure

First, the MAES department heads at each university were contacted to ask for their collaboration. After informing them about the project’s objective and characteristics, permission was obtained from the universities included in the study. Subsequently, the students were contacted via email to request their collaboration and to complete the online form. In this communication, they were informed about the study objective, the anonymous nature of their responses, the way to complete the scales, and to make them aware that they could leave the study at any time. All the participants gave their informed consent before completing the questionnaire. The study was carried out following the Declaration of Helsinki and the protocol was approved by the Bioethics Committee of the University of Almería (Ref:UALBIO2021/009).

### 2.4. Data Analysis

SPSS v.29 software was used to calculate the descriptive statistics, correlations, and Student’s *t*-test to determine the mean difference according to gender. The internal consistency of each scale was estimated using McDonald’s omega (ω), with reliability values > 0.70 being considered acceptable [44]. A CFA of each scale was carried out using AMOS v.29 software. Regarding the main analyses, a SEM of the latent variables was calculated in two steps, again using AMOS v.29 and following Kline [45]; this was caried out to study the predictive relationships between academic self-concept and teaching identity, analysing the mediating role of the SEE. As a first step, the robustness of the bidirectional relationships between the SEM variables (the measurement model) was evaluated, while in the second step, the predictive effects between the dimensions were studied. The SEM was controlled by the home university. The SEMs were evaluated taking into account the goodness of fit: CFI, TLI, RMSEA with their 90% confidence interval (CI), SRMR, and χ^2^/df. The following values were considered acceptable: χ^2^/df ratio < 5.0; CFI and TLI > 0.90; and RMSEA and SRMR < 0.08 [46,47]. The maximum likelihood method with bootstrapping was used for 5000 resamplings due to the lack of multivariate normality in the multigroup SEM (Mardia’s coefficient, men = 7.41, *p* < 0.001; women = 12.44, *p* < 0.001) [45]. In addition, the indirect effects and their 95%CI were estimated using the bootstrapping technique, with the indirect effects being considered significant (*p* < 0.05) if their 95%CI did not include zero [48]. Finally, following Domínguez-Lara [49], the explained variance (R^2^) was considered as the effect size (ES), considering values < 0.02 small, close to 0.13 medium, and >0.26 large [50]. The CIs (95%) of R^2^ were also used to ensure that no value was less than the minimum required for interpretation purposes (0.02).

## 3. Results

### 3.1. Participants

A total of 478 MAES students participated. Each was enrolled in the PE specialty at one of eight Andalusian universities. They were aged between 21 and 47 years old (M = 27.09; SD = 6.32). Since we were studying the variables according to gender, two responses were not used because the answer given was ‘other gender’ (0.4%); therefore, we considered a total of 476 responses (55.8% men; 44.8% women). The data collection fieldwork was conducted in May 2022. There are no missing values in the included data.

### 3.2. Preliminary Results

Table 1 shows the descriptive statistics of the different variables taking into account the gender of the trainee teachers, as well as the results of the Student’s *t*-test. As one can observe, Table 1 indicates statistically significant differences in academic effort. Furthermore, for the other two variables, men obtained higher mean values in SEE and in teaching identity. No statistically significant differences were found in teaching identity or in SEE.

As shown in Table 1, both men and women positively correlate the two dimensions of academic self-concept and SEE. In addition, one can see that academic confidence does not correlate with teaching identity among women, whereas among men, it correlates positively. Furthermore, high correlation values were found between the SEE and academic confidence in future male teachers, whereas in future female teachers, the values were low.

### 3.3. Main Results

**Figure 2:**
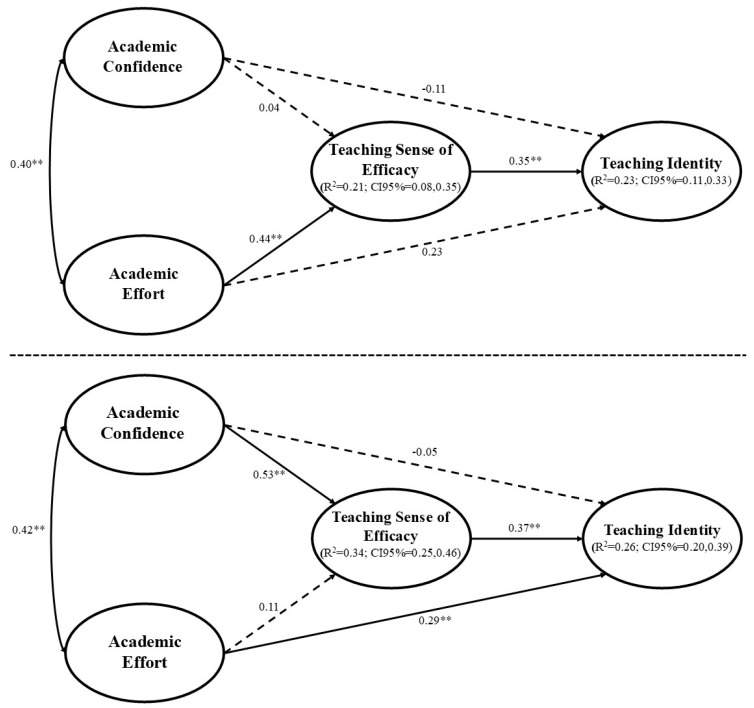
Predictive relationships of academic confidence and academic effort to teaching identity mediated by the sense of teaching efficacy in women (**top**) and men (**bottom**). Note: ** *p* < 0.01;. R^2^ = Explained variance; CI = Confidence interval. The 95%CI is reported in parentheses. The dotted arrows represent the non-significant relationships.

Since significant differences were found according to gender, a multigroup SEM analysis was carried out (i.e., male trainee teachers, female trainee teachers). In Step 1, the multigroup SEM presents acceptable goodness-of-fit indices: χ^2^/df = 2.12, *p* < 0.001; CFI = 0.937; TLI = 0.924; RMSEA = 0.049 (90%CI = 0.043; 0.054); and SRMR = 0.057, as it also does in Step 2: χ^2^/df = 2.12, *p* < 0.001; CFI = 0.937; TLI = 0.924; RMSEA = 0.049 (90%CI = 0.043; 0.054); and SRMR = 0.057. The SEM was controlled by the participants’ home university. The explained variance reached 23% and 26% for teaching identity in women and men, respectively, and 21% and 34% for SEE in women and men, respectively (Figure 2). The different predictive relationships between the dimensions of academic self-concept (i.e., confidence and effort), SEE, and teaching identity, as well as the mediating effect of SEE, are shown in Figure 2, Table 2 and Table 3, differentiating the results between women and men.

In the SEM, one can verify the different effects that the perception of academic confidence and effort have between female and male pre-service teachers, establishing different development pathways towards teaching identity and highlighting the mediating role of SEE. Among the women, academic effort is a direct, positive and significant predictor (*p* < 0.001) of SEE whereas confidence is not significantly related to SEE (*p* = 0.654). In the case of men, it is confidence that has a direct, positive and statistically significant relationship to SEE (*p* < 0.001), while effort does not present a significant relationship (*p* = 0.085). Likewise, among women, neither confidence (*p* = 0.159) nor effort (*p* = 0.054) have a significant direct relationship to teaching identity. On the other hand, among male students, it seems that academic effort is more relevant in developing teaching identity, since these variables are directly and significantly related (*p* = 0.002); however, nor is academic confidence a direct predictor of teaching identity among men (*p* = 0.592). On the other hand, SEE is important in increasing teaching identity, both in women (*p* < 0.001), and in men (*p* < 0.001). In addition, the mediating effect of SEE should be noted since, among women, it serves to increase the development of professional identity (β = 0.16; *p* < 0.001). In the case of men, SEE mediation serves to make confidence indirectly increase professional identity (β = 0.19; *p* = 0.002). Finally, Figure 2 also presents the CIs (95%) of R^2^, confirming that they can be considered as ES measures.

## 4. Discussion

The present research had two main objectives. The first objective was to identify the differences according to gender in the students’ perceptions of their academic self-concept, SEE, and teaching identity. The second objective was to analyse the differentiated role of academic self-concept on SEE and teaching identity, taking into account the gender of the pre-service teachers. The main results of this study revealed that (i) there were differences in academic effort based on the gender of the pre-service teachers (i.e., men and women); (ii) academic effort indirectly favoured teaching identity in women teachers in initial training; and (iii) academic confidence indirectly predicted the teaching identity of teachers in initial training.

### 4.1. Gender Differences

Regarding the first objective, our results showed no gender-based differences in the students’ perceptions of their SEE and teaching identity amongst the pre-service PE teachers. However, the women reported significantly higher perceptions of academic effort than the men. In line with our results, Liu et al. [38] found gender-based differences in academic self-concept, showing a high effort value in the girls. Even so, with the exception of the Liu et al. [38] study, they do not completely coincide with the few existing studies that have assessed gender differences. In contrast to our findings, Belfi et al. [7] did not report conclusive results regarding possible gender-based differences in academic self-concept. However, it should be noted that all the above-mentioned studies measured the perception of academic self-concept in children and adolescents and are therefore not comparable to trainee teachers.

### 4.2. Different Pathways

With regard to the second objective, our results showed that SEE mediated the relationship between academic self-concept and teaching identity. These results are consistent with the scientific evidence in the teacher training context [24], indicating that self-concept can stimulate internal resources during the student training process. In this sense, although both relationships were statistically significant, the relationships between academic self-concept and teacher identity differed based on the gender of the trainee teachers. On the one hand, effort had direct effects on teaching identity in men, while self-concept (i.e., confidence and effort) had no direct effect on teaching identity in the women. These differences can be explained for different reasons. Firstly, as the preliminary analyses have shown, male pre-service teachers present significant differences in one of the academic self-concept dimensions compared to the female pre-service teachers. Another possible explanation could be due to the fact that the men have a greater predominance of academic self-concept, as reflected in some of the previous research [38]. In this way, academic effort would play an explanatory role in intrapersonal aspects [23]; therefore, in the teacher training context, it would be more relevant for developing teaching behaviours, such as the creation of professional identity.

Continuing with the hypothesized model, indirect effects mediated by teacher efficacy followed different pathways according to the students’ gender. On the one hand, the indirect effects of effort on teaching identity mediated by SEE were significant for the women, whereas they were not significant for the men. Conversely, confidence mediated by SEE predicted teaching identity in the men whereas this relationship was not significant in the female trainee teachers. A possible justification for this inconsistency might lie in the fact that self-concept has differential contextual implications according to the students’ perception of professional efficacy, in the context of teacher training [15]; that is to say, in men, the distinctive role of academic confidence would significantly help develop their teaching competence and consequently increase their professional identity, whilst in girls, this teaching identity development would occur because of a more developed level of effort.

### 4.3. Limitations and Future Lines of Study

Different strengths can be appreciated in these research results. First, our study is one of the few to examine a gender-based distinction in the professional identity of pre-service teachers. In addition, and more specifically, this study examined how the intrapersonal resources of teachers differed according to the gender of the pre-service teachers, thus making it possible to adjust specific didactic strategies to intervene in the training process. Despite the relevance of these results, the present research has certain limitations. First, the data used should be interpreted with caution because of the non-probabilistic sampling method. Second, despite the fact that the SEM was based on the SCT postulates and the theoretical model of [23], it is impossible to make causal inferences due to the cross-sectional nature of the study. Therefore, future studies should undertake longitudinal analyses that evaluate the causality of the variables during the training process. Finally, this research examined intrapersonal resources according to the students’ gender. Future studies should measure socio-contextual variables according to gender differentiation in order to determine how specific teaching strategies influence teacher training.

### 4.4. Practical Implications

These research results have relevant practical implications for teacher trainers in Physical Education. On the one hand, the findings reveal that, in men, the teacher should emphasize behaviours that develop confidence through teaching efficacy and increase teaching identity. To this end, the teacher trainer should be aware of previous behaviours and be able to implement strategies that develop confidence. As an example, it is necessary for the teacher trainer to implement practices such as meaningful feedback, error acceptance as part of the learning process, and to adjust the level of demand [51,52,53]. Furthermore, given the main findings of this study, teacher educators should establish teaching practices that favour the effort of trainee teachers. As an example, it is recommended that motivation and autonomy are fostered, perseverance and work are valued, and that personal satisfaction is focused upon [53,54]. Similarly, using pedagogical models such as service-learning, which can integrate specific didactic strategies, would be highly recommended for PE teacher trainers [55]. In this way, understanding how the academic image of trainee teachers influences the creation of their professional identity could be very useful for developing analytical strategies for teacher training programmes.

## 5. Conclusions

The present research provides evidence from the teacher training field, demonstrating that higher effort values were found in Physical Education trainee teachers. In addition, it has shown that academic self-concept affects teaching efficacy differently according to the gender of the trainee teachers. Thus, the results of the present study suggest that, although both dimensions of self-concept were related to teaching identity, in boys teaching efficacy mediated the relationship between academic confidence and teaching identity, whereas in girls, it was academic effort that did so. Therefore, the results show that teacher educators should vary their teaching strategies between the two sexes to develop the students’ teaching identity.

## Figures and Tables

**Table 1:** Descriptive statistics and mean differences between pre-service teachers.

	Women (*n* = 214)		Men (*n* = 262)		Mean Difference				Correlations			
Variables	M	SD	M	SD	*Dif*	*t* (gl)	*p*-Value	*d*	1	2	3	4
1. Effort	5.55	1.14	5.19	1.24	0.36	3.27 (474)	<0.001	0.30	-	0.26 **	0.37 **	0.30 **
2. Confidence	6.00	0.96	5.93	0.86	0.07	0.82 (474)	0.412	0.08	0.32 **	-	0.16 *	0.13
3. SEE	7.40	1.11	7.48	1.08	−0.07	−0.78 (474)	0.437	0.07	0.27 **	0.46 **	-	0.44 **
4. Teaching Identity	4.14	0.80	4.25	0.70	−0.12	−1.67 (426) ^a^	0.095	0.15	0.33 **	0.18 **	0.42 **	-

Note: SEE = Sense of teaching efficacy; SD = Standard deviation; *Dif* = Mean differences; *d* = Cohen’s d; ^a^ = Levene’s test is significant, the variances are not equal. Numbers above the diagonal show the correlations for female pre-service teachers. Numbers below the diagonal show the correlations for male pre-service teachers. ** *p* < 0.01; * *p* < 0.05.

**Table 2:** Estimation of significant standardized parameters and statistics of the mediation model (women).

Independent Variable	Dependent Variable	Mediator	β	SE	95%CI	
					Upper	Lower
Direct effects						
Confidence	SEE		0.04	0.09	−0.12	0.21
Effort	SEE		0.44 **	0.09	0.25	0.62
Confidence	Teaching Identity		−0.11	0.07	−0.25	0.05
Effort	Teaching Identity		0.23	0.08	0.01	0.45
SEE	Teaching Identity		0.36 **	0.06	0.18	0.52
Indirect effects						
Confidence	Teaching Identity	SEE	0.01	0.03	−0.05	0.08
Effort	Teaching Identity	SEE	0.16 **	0.03	0.07	0.24

Note. β = Estimation of standardized parameters; SEE = Sense of teaching efficacy; SE = standard error; 95%CI = 95% confidence interval; Lower = 95%CI lower limit; Upper = 95%CI upper limit; ** *p* < 0.01. Men’s scores are reported in parentheses.

**Table 3:** Estimation of significant standardized parameters and statistics of the mediation mode (men).

Independent Variable	Dependent Variable	Mediator	β	SE	95%CI	
					Upper	Lower
Direct effects						
Confidence	SEE		0.53 *	0.08	0.40	0.64
Effort	SEE		0.11	0.07	0.02	0.24
Confidence	Teaching Identity		−0.05	0.08	−0.24	0.14
Effort	Teaching Identity		0.29 **	0.05	0.13	0.46
SEE	Teaching Identity		0.37 **	0.06	0.19	0.54
Indirect effects						
Confidence	Teaching Identity	SEE	0.19 **	0.05	0.08	0.31
Effort	Teaching Identity	SEE	0.04	0.01	0.01	0.09

Note. β = Estimation of standardized parameters; SEE = Sense of teaching efficacy; SE = standard error; 95%CI = 95% confidence interval; Lower = 95%CI lower limit; Upper = 95%CI upper limit; ** *p* < 0.01; * *p* < 0.05.

## Data Availability

The data presented in this study are available on request from the corresponding author. The data are not publicly available due to privacy.
